# The role of sonication in the microbiological diagnosis of implant-associated infections – the experience of the National Institute for Infectious Diseases “Prof. Dr. Matei Balş”, Bucharest

**DOI:** 10.1186/1471-2334-13-S1-O35

**Published:** 2013-12-16

**Authors:** Raluca Mihăilescu, Daniela Tălăpan, Olga Dorobăț, Alexandru Rafila, Emilia Capraru, Daniela Munteanu, Anca Streinu-Cercel, Oana Streinu-Cercel, Vlad Predescu, Florian Purghel, Cătălin Cârstoiu, Razvan Ene, Dana Mihaela Jianu, Cristina Popescu, Victoria Aramă, Adrian Streinu-Cercel

**Affiliations:** 1 National Institute for Infectious Diseases “Prof. Dr. Matei Balş”, Bucharest, Romania; 2 Carol Davila University of Medicine and Pharmacy, Bucharest, Romania; 3 Department of Orthopedics and Traumatology, Emergency Clinical Hospital “Sf. Pantelimon”, Bucharest, Romania; 4 Department of Orthopedics and Traumatology, Emergency Clinical Hospital “Prof. Dr. Bagdasar-Arseni”, Bucharest, Romania; 5 Department of Orthopedics and Traumatology, Emergency University Hospital, Bucharest, Romania; 6 ProEstetica Medical Center, Bucharest, Romania

## Background

Sonication optimizes the microbiological diagnosis of implant-associated infections. It is recommended in international guidelines of Infectious Diseases, but not yet worldwide accessible. In Romania, sonication of medical implants was first introduced in 2012 in the National Institute for Infectious Diseases “Prof. Dr. Matei Balş”, Bucharest.

## Methods

We present the status report on sonication of medical implants in Bucharest, Romania during 2012 and 2013.

## Results

We processed 10 joint prostheses sent by three departments of orthopedics and 7 breast implants from one center of plastic surgery in Bucharest. Sonication of the prostheses revealed either monomicrobial infections: *Parvimonas micra*, *Burkholderia cepacia* (n=4), *Serratia marcescens*, *Staphylococcus warneri* or polymicrobial infections: *Burkholderia cepacia* and *Staphylococcus aureus* (n=2); *Pseudomonas aeruginosa*, *Klebsiella pneumoniae* ESBL+ and *Enterococcus faecium*. Mammary implants were screened, but all were found culture-negative.

We also present the first case of sonication – a polymicrobial infection with methicillin resistant *Staphylococcus aureus* and *Burkholderia cepacia*. *B cepacia* was susceptible to piperacillin ± tazobactam, ceftazidime, meropenem, co-trimoxazole. The patient received vancomycin i.v. 500 mg Q6h + co-trimoxazole 400/80 mg 2tb Q8h p.o. + ceftazidime 2g Q8h i.v. for 6 weeks and was then switched to ciprofloxacin 750 mg Q12h + co-trimoxazol 2tb Q8h p.o. for a total of 5 months. As CRP remained around 25 mg/L, a synovial puncture was requested. The synovial fluid was culture-negative and the synovial leucocyte count was within the normal range. Subsequently, revision arthroplasty was performed, with favorable outcome.

## Conclusion

Sonication is a key tool for the accurate microbiological diagnosis of implant-associated infections, dislodging the biofilm-forming microbes from the implant surface. It is helpful even if the infection is polymicrobial. It also revealed nosocomial infections which needed further epidemiological measures. A multidisciplinary approach (microbiology, infectious diseases and surgery) is essential for the management of implant-associated infections.

